# Comparison of customized vacuum sealing drainage and vacuum sealing drainage in the treatment of diabetic foot ulcers: a retrospective analysis

**DOI:** 10.1186/s13018-023-04298-z

**Published:** 2023-10-27

**Authors:** Qingling Chen, Minting Lu, Xueyan Liu, Yanmei Yu, Jiaying Li

**Affiliations:** 1 Department of Endocrinology, First Affiliated Hospital of Jinan University, Guangzhou, 510632 China; 2 School of Nursing, Jinan University, Guangzhou, 510632 China; 3 Department of Endocrinology and Metabolism, The First Affiliated Hospital of Jinan University, Guangzhou, 510632 China

**Keywords:** Diabetic foot ulcers, Vacuum sealing drainage, Customized vacuum sealing drainage, Retrospective analysis

## Abstract

**Background:**

The prevalence of diabetic foot ulcers, a common, more serious chronic diabetes-related complication, is increasing. Vacuum sealing drainage (VSD) constitutes an effective adjunctive treatment for diabetic foot ulcers. Factors, such as poor glycemic control, ischemia, and infection prolong wound healing time, and VSD products are expensive and unaffordable for many patients.

**Objective:**

To compare the use of customized VSD and customized VSD in the treatment of diabetic foot ulcer.

**Method:**

This retrospective study included 83 patients with diabetic foot ulcers in customized VSD (*n* = 44) and VSD (*n* = 39) groups. Baseline data, efficacy after 14 days, total treatment efficiency, final outcome (28 days after treatment, healing rate), average treatment cost, and hospitalization (days) of the two groups were compared. Factors affecting wound healing were analyzed.

**Results:**

No significant intergroup differences in the baseline data were detected (VSD vs. customized VAD, *p* > 0.05). Treatment efficacy was higher in the customized VSD group than in the VSD group after 14 days (*p* < 0.05), although total treatment efficiency in both groups reached 100%. The final outcome in the customized VSD group was better (vs. VSD group, *p* < 0.05), and the wound healing rate was higher than in the VSD group (66.7% vs. 33.3%). The mean treatment cost and hospital days were greater in the VSD group (vs. customized VSD group; *p* < 0.05). Factors affecting wound healing include age, Wagner classification, HDL-C, and fasting C-peptide. Younger age, low Wagner classification grade, low HDL-C level, and high fasting C-peptide contribute to higher healing rate,

**Conclusion:**

Efficacy and final outcome of customized VSD were better than that of VSD; the customized VSD device is simple and convenient to operate, and enables cost-effective treatment.

## Introduction

The prevalence of diabetes mellitus in the worldwide is gradually increasing [1]. Diabetic foot is one of the common and more serious chronic complications of diabetes mellitus, and is characterized by the infection, ulceration and (or) deep tissue destruction of the foot and (or) lower extremity that is caused by diabetic neuropathy and/or varying degrees of peripheral vascular disease [2]. According to the International Diabetic Foot Working Group [1], on average, one patient with diabetes undergoes limb amputations every year. With the associated high recurrence, disability, and mortality rates [3, 4], diabetic foot ulcers have seriously affected the quality of life of patients, and imposed enormous pressure and heavy burdens on families and the public health system.

Studies have indicated that surgery is important to mechanically stabilize and harmonize the foot for long-term off-loading and food-protection [5]. In addition, combining surgery and antibiotic therapy seems to be more effective compared to each one alone [5, 6]. Vacuum sealing drainage (VSD) is an effective adjunctive treatment for diabetic foot ulcers that can improve local blood circulation, promote growth of granulation tissue and wound healing, reduce the exudation of tissue fluids, maintain wound moistness, and reduce the infection rate of the wound; furthermore, VSD can, to some extent, alleviate the diabetic foot ulcer-related pain of patients. For example, it has been shown that VSD is a more effective therapy and is associated with a greater decrease in wound size and shorter time to wound healing, compared to the conventional method [7]. The acceleration of the wound healing process was attributed to enhancing the inflammatory response and promoting granulation and angiogenesis in diabetic foot ulcers [7]. It is worth to mention that there are limitations on the use of VSD. The main limitation to applying VAC occurs when attempting to maintain an airtight seal over irregular surfaces surrounding a wound [8]. VAC may also make Infectious necrotic tissue which may be adsorbed on spongy suction materials with a negative pressure, resulting in blockage [9]. If the spongy materials have no antibacterial properties, secondary infection may happen [10]. Therefore, to overcome shortcomings of VSD, it is necessary to introduce customized VSD into the treatment of diabetic foot ulcers.

Customized VSD technique is a new treatment method of covering or filling the wound and soft tissue defects with a hydrocolloid dressing that contains a flow tube, which is covered with a sterile gauze dressing, closed with a biological semi-permeable membrane to ensure an airtight seal, and finally passing the drainage tube through the negative pressure source to promote wound healing by controlled negative pressure and simultaneous saline flushing.

This study was conducted with an aim to investigate the clinical usefulness of customized VSD for wound healing of diabetic foot ulcers and, thereby, provide a simple, economical, and effective method for the clinical treatment of patients with diabetic foot ulcers.

## Methods

### Overview

This retrospective data analysis was performed in a convenience sampling-selected cohort of 83 participants with diabetic foot ulcers who were hospitalized and received either VSD or customized VSD carried out by Doctors in the Department of Endocrinology and Metabolism of the First Hospital Affiliated of Jinan University from September 2019 to December 2020. According to the treatment method, the study cohort was divided into the VSD group (*n* = 45) and the customized VSD group (*n* = 49) that had comparable clinical characteristics without statistically significant difference (*p* > 0.05; Table 1).

**Table 1:** Comparison of clinical characteristics between the two groups of participants

Characteristic	Customized VSD group (*n* = 44)	VSD group (*n* = 39)	t / Z /χ^2^ value	*P* value
Sex			0.479	0.489
Man	27 (61.4%)	21 (53.8%)		
Women	17 (38.6%)	18 (46.2%)		
Age (years)	66.34 ± 11.51	67.18 ± 12.12	0.522	0.747
Duration of diabetes mellitus, years			1.248	0.212
≤ 5	12 (27.3%)	8 (20.5%)		
6–10	18 (40.9%)	14 (35.9%)		
11–15	3 (6.8%)	5 (12.8%)		
16–20	11 (25%)	7 (17.9%)		
> 20	0 (0%)	5 (12.8%)		
BMI (kg/m^2^)	22.44 (20.85–24.12)	22.80 (21.10–23.80)	0.105	0.916
FPG (mmol/L)	10.55 (7.80–13.70)	8.85 (6.74–12.34)	1.306	0.192
HbA1c (%)	8.55 (7.00–10.60)	9.60 (7.80–10.60)	1.209	0.226
Fasting C-peptide (ug/L)	2.31 (1.31–3.36)	1.83 (0.89–2.63)	1.811	0.07
TC (mmol/L)	4.18 ± 1.01	4.18 ± 1.24	0.013	0.989
TG (mmol/L)	1.30 (0.91–1.55)	1.35 (1.00–1.79)	0.767	0.443
HDL-C (mmol/L)	0.88 (0.80–1.16)	0.85 (0.68–1.12)	0.612	0.541
LDL-C (mmol/L)	2.48 ± 0.69	2.38 ± 0.83	0.609	0.544
Hemoglobin (g/L)	123 (110–134.25)	117 (102–135)	0.717	0.474
Wagner Grading			3.556	< 0.001
Level 2	18 (40.9%)	6 (15.4%)		
Level 3	22 (50%)	17 (43.6%)		
Level 4	4 (9.1%)	16 (41.0%)		

BMI: body mass index; FPG: fasting plasma glucose; TC: total cholesterol; TG: triglycerides; HDL-C: high-density lipoprotein cholesterol; LDL-C: low-density lipoprotein cholesterol; HbA1c: glycated hemoglobin

### Inclusion and exclusion criteria

The inclusion criteria included (1) meeting the diagnostic criteria for diabetic foot established by WHO in 1999; (2) time since wound formation > 1 month; and (3) diabetic foot ulcers of Wagner classification grades 2–4. The exclusion criteria included: (1) severe complications and multiple organ failure; (2) treatment with immunosuppressive drugs, glucocorticoids, and chemotherapy; and (3) hypoproteinemia (serum albumin < 25 g/L) and severe anemia (hemoglobin < 70 g/L) despite appropriate symptomatic treatment.

### Ethics approval

This study was approved by the Ethics Committee of the First Hospital of Jinan University (KYk-2022025). All participants signed an informed consent form for study participation.

### Grouping and treatment procedures

#### Customized VSD group

Under local anesthesia, the necrotic tissue on the wound surface was fully removed, and complete hemostasis was ensured after debridement; holes were cut along the side of a disposable suction tube according to the size of the wound, and a scalp needle hose was placed in the middle of the suction tube; thereafter, a lipid hydrocolloid dressing was placed on the wound and covered with a layer of sterile gauze; the surrounding skin was dried, and a 3 M transparent film (3 M Company, USA) was applied to seal the entire wound surface, and negative pressure was maintained at 40–60 kPa after testing to ensure that there was no air leakage; then, saline was infused through the scalp needle connector to ensure continuous flushing during treatment. Dressings were changed depending on the amount of exudate, and necrotic tissues were excised in each dressing change. Other concomitant therapeutic measures (e.g., anti-infection, blood sugar control, neuronal nutrition, improvement of microcirculation, restoring patency of blood vessels, etc.) were continued as relevant.

#### VSD group

In the VSD group, the treatment involved adequate removal of necrotic tissue from the wound surface, thorough post-debridement hemostasis, and use of the VSD single-use negative pressure drainage wound protection material (drainage tube set). Dressings were changed according to the amount of exudate, and necrotic tissues were excised in each dressing change. Other treatment measures were continued similarly as in the customized VSD group.

### Outcome indicators

The treatment outcome (after 14 days), total treatment efficiency, final outcome (after 14 days), average hospitalization cost, and length of hospitalization (days) were compared between the two groups. Efficacy was determined based on the following criteria: (1) *cure*: the wound had completely healed, and exudation, redness, swelling, and pain had disappeared completely; (2) *improvement*: the patient's wound exudates had decreased, there was new granulation, the skin around the wound was growing normally, and the wound area was reduced by more than half of that at treatment initiation; (3) *ineffective*: the patient's wound was not fresh, for example, there was heavy exudation, absence of healthy granulation, and the wound area reduction was less than half of that at treatment initiation. The total effective rate of treatment was equal to the sum of the healing rate and the improvement rate. The final regression status was classified as complete healing, significant effect (wound healing area of three-fourth or more of that at treatment initiation, significant reduction of exudation, redness, swelling, and pain), amputation of the toe, amputation above the toe, and death.

### Statistical analysis

Statistical processing and analysis were performed using IBM SPSS 26.0. The count data were described as the frequency and composition ratio, and the Chi-square test was used for intergroup comparison. The measurement data with normal distribution were expressed as the mean and standard deviation, whereas data with skewed distribution were expressed as median (interquartile range); Independence t test was used for the comparison of data that conformed to normal distribution with homogeneous variance; the rank sum test was used for the comparison of variables that did not follow normal distribution or for ordered categorical variables. Pearson and Spearman correlation analysis tests were used for correlation analysis. *p* < 0.05 indicates statistical significance.

## Results

### Clinical characteristics of participants

A total of six patients in VSD group and five patients in customized VSD group were dropped mainly because of the need of immunosuppressive drugs or chemotherapy or onsite of severe complications during the study, leading to an attrition rate was 11.7%.

The VSD and customized VSD groups included 21 men and 18 women (mean age 67.18 ± 12.12 years) and 27 men and 17 women (mean age 66.34 ± 11.51 years), respectively. There was no significant intergroup difference in sex, age, BMI, disease duration, and HbA1c (*p* > 0.05) and both the groups had comparable clinical data (Table 1).

### Comparison of treatment outcome (after 14 days), final outcome (after 28 days), average hospitalization cost, and hospitalization stay (days) in both groups

Patients in the customized VSD group had a slightly better treatment effect than those in the VSD group after 28 days, although the total treatment efficiency was 100% in both groups. The wound healing rate of patients in the customized VSD group (66.7%) was higher than that of patients in the VSD group (33.3%). The mean hospitalization costs and hospitalization stay (days) of patients in the VSD group were more than those of patients in the customized VSD group (Table 2).

**Table 2:** Comparison of the treatment effect (14 days after treatment), final outcome (28 days after treatment), average hospitalization cost, and hospitalization days between the two groups

	Customized VSD Group (*n* = 44)	VSD Group (*n* = 39)	Z value	*P* value
Treatment effect			2.158	0.031
Ineffective	0 (0%)	0 (0%)		
Improvement	39 (88.6%)	39 (100%)		
Healing	5 (11.4%)	0 (0%)		
Final outcome			4.036	< 0.001
Death	0 (0%)	0 (0%)		
Amputation above the toe	0 (0%)	2 (5.1%)		
Amputation of the toe	3 (6.8%)	14 (35.9%)		
Proven effectiveness	1 (2.3%)	3 (7.7%)		
Fully healed	40 (90.9%)	20 (51.3%)		
Average hospitalization days	18 (11–28)	28 (20–50)	3.351	0.001
Average hospitalization cost (RMB)	17,504 (12,571–22,563)	52,705 (30,109–91309)	5.877	< 0.001

### Factors affecting wound healing of diabetic foot ulcers

Univariate analysis demonstrated that age, Wagner classification, fasting C-peptide level, HDL-C, and treatment methods had significant effect on the healing of diabetic foot ulcers (*p* < 0.05; Table 3).

**Table 3:** Factors affecting the healing of diabetic foot ulcers

Groups	Non-healing group (*n* = 23)	Healing group (*n* = 60)	*t*/*Z*/*χ*^2^ value	*P* value
Age (years)	72.91 ± 11.23	64.37 ± 11.13	3.125	0.002
Sex			2.688	0.101
Man	10 (43.5%)	38 (63.3%)		
Women	13 (56.5%)	22 (36.7%)		
Duration of diabetes mellitus, years			1.561	0.118
Wagner Grading			5.645	< 0.001
Level 2	2 (8.7%)	22 (36.7%)		
Level 3	3 (13.0)	36 (60.0%)		
Level 4	18 (78.3%)	2 (3.3%)		
Treatment method			16.207	< 0.001
Customized VSD	4 (17.4%)	40 (66.7%)		
VSD	19 (82.6%)	20 (33.3%)		
FPG (mmol/L)	8.94 (7.26–12.03)	10.0 (7.0–13.4)	0.605	0.545
HbA1c (%)	8.6 (7.2–10.5)	9.10 (7.53–11.05)	0.509	0.611
BMI (kg/m^2^)	23.0 (21.43–23.80)	22.38 (21.01–24.05)	0.290	0.772
TC (mmol/L)	4.53 ± 1.12	4.05 ± 1.10	1.785	0.078
TG (mmol/L)	1.37 (1.15–1.91)	1.28 (0.91–1.62)	1.201	0.230
HDL-C (mmol/L)	0.96 (0.83–1.28)	0.86 (0.68–1.04)	2.036	0.042
LDL-C (mmol/L)	2.52 ± 0.71	2.40 ± 0.78	0.626	0.533
SBP (mmHg)	138 (125–146)	138 (128–144.75)	0.127	0.899
DBP (mmHg)	75 (70–86)	75 (68.25–80)	1.076	0.282
Hemoglobin (g/L)	119 (108–135)	119.5 (108–133.5)	0.158	0.875
Fasting C-peptide (ug/L)	1.21 (0.83–2.30)	2.31 (1.55–3.28)	3.465	0.001

BMI: body mass index; FPG: fasting plasma glucose; TC: total cholesterol; TG: triglycerides; HDL-C: high-density lipoprotein cholesterol; LDL-C: low-density lipoprotein cholesterol; HbA1c: glycated hemoglobin; SBP: systolic blood pressure; DBP: diastolic blood pressure

### Correlation analysis of age, Wagner classification, HDL-C, fasting C-peptide, and wound healing

Correlation analysis results showed that age (*p* = 0.002), Wagner classification (*p* < 0.001), and HDL-C (*p* = 0.041) were negatively correlated with wound healing, whereas fasting C-peptide level (*p* < 0.001) was positively correlated with wound healing (Table 4).

**Table 4:** Correlation analysis of age, Wagner classification, HDL-C, fasting C-peptide, and healing

Item	Value	*r*/*r*_s_ value	*P* value
Age	72.91 vs 64.37	− 0.328	0.002
Wagner	2 vs 22; 3 vs 36; 18 vs 2	− 0.623	< 0.001
HDL-C	0.96 vs 0.86	− 0.225	0.041
Fasting C-peptide	1.21 vs 2.31	0.383	< 0.001

HDL-C: high-density lipoprotein cholesterol; r: Pearson's correlation coefficient for continuous variables; r_s_: Spearman's correlation coefficient

## Discussion

Diabetic foot ulcers are one of the most serious chronic complications of diabetes and are a major cause of disability and death in patients with diabetes [3], and are mainly caused by peripheral neuropathy and vascular disease [11]. Endogenous changes, such as neurological, vascular, immune, and metabolic changes, and exogenous factors, such as infection, trauma, and pressure, jointly lead to the occurrence of diabetic foot ulcers that are difficult to heal, and the interaction between the causative factors forms a complex pathophysiological process in diabetic foot ulcers [12, 13]. Therefore, the treatment of diabetic foot involves multiple disciplines and requires systematic and comprehensive treatment, including glycemic control, surgical debridement, revascularization, decompression therapy, and supportive therapy, among which control of wound infection and promoting tissue repair are key to preventing amputation or for reducing the plane of amputation [14].

Studies revealed that surgery or combination of surgery with antibiotic therapy plays a very important role in stabilizing and harmonizing the foot for long-term off-loading and foot-protection [5, 6]. Besides surgery, other like knowledges have also been explored in the treatment of diabetic foot ulcers. For example, negative pressure wound therapy (NPWT) is widely applied for various acute and chronic wounds, such as diabetic foot ulcers, because of effects that improve wound drainage, increase vascular perfusion, and promote growth of granulation tissue [14, 15]. One of the key technologies of NPWT is vacuum sealing drainage (VSD) [16], which accelerates the wound healing process by facilitating the restoration of normal tissue morphology, infection control, enhanced inflammatory response, and promotion of wound granulation and angiogenesis [7]. Several studies [7, 15, 17–25] have shown that, compared with traditional diabetic foot wound treatment modalities (e.g., routine drug changes, etc.), VSD can significantly improve the wound healing rate, shorten wound healing time, reduce amputation rate, etc., and reduce the number of drug changes, which helps to reduce the workload of healthcare workers.

Nonetheless, the high cost of VSD product retrieval increases the average cost of treatment and imposes a heavy financial burden on patients and families, whereas customized VSD is easy to operate, more economical, and constitutes a safe and effective treatment. In this study, we used a customized VSD technique to treat patients with diabetic foot ulcers that is a new treatment method of covering or filling the wound and soft tissue defects with a hydrocolloid dressing that contains a flow tube, which is covered with a sterile gauze dressing, closed with a biological semi-permeable membrane to ensure an airtight seal, and finally passing the drainage tube through the negative pressure source to promote wound healing by controlled negative pressure and simultaneous saline flushing. The customized VSD technique used in this study recruits the following mechanisms to promote the healing of diabetic foot ulcers: (1) continuous expulsion of exudate, free radicals, cytokines, and other inflammatory mediators from the wound surface to accelerate wound healing; (2) the negative pressure environment created in the wound tissue can improve blood flow, promote the removal of harmful substances, and promote the growth of granulation tissue; (3) reducing the number of bacteria on the wound surface; and (4) the pressure generated by the negative pressure device can lead to effective wound healing. The use of negative pressure suction in patients with diabetic foot ulcers together with other comprehensive treatments can significantly accelerate the healing of the wound and has a positive effect on preventing inflammation spread and osteomyelitis.

Studies have shown that total contact casts (TCCs) significantly reduce pressure on wounds and have been shown to heal between 73 and 100% of all diabetic foot wounds treated with them [26]. But TCCs are difficult and time-consuming to apply. Our study showed that customized VSD group showed a healing rate of 90.9% (VSD group showed a healing rate of 51.3%), which is comparable to the efficacy of TCCs. Since customized VSD is easy to be applied, it is of great significance in the treatment of diabetic foot ulcers.

Yang et al. reported that mean healing rate of the ulcers in the VSD group was significantly higher than that in the control group (35.23 ± 2.87% vs 28.78 ± 1.09%, *P* = 0.017) [7]. Another study demonstrated that the ulcer healing rate in percutaneous endovascular angioplasty combined with negative pressure closed drainage (PTA-VSD) group at 180 days post-surgery was significantly greater than that of the percutaneous endovascular angioplasty combined with depuration (PTA-UD) group (52% vs. 12%) (*P* = 0.002, < 0.05) [27]. The results of this study also showed that the treatment of diabetic foot ulcers using a customized VSD technique was slightly more effective than treatment with the VSD technique, and that the final healing of foot ulcer wounds was better in the novel customized VSD group than in the VSD group (66.7%% vs. 33.3%). Furthermore, the customized VSD technique was easier and more economical (lower average cost of treatment and fewer average hospital days), which could reduce the financial burden of the patients. In addition, this study included a univariate analysis to identify clinical factors that influence the final outcome of diabetic foot ulcer healing, and the results showed that age, Wagner classification, HDL-C, and fasting C-peptide levels influence foot ulcer wound healing. With increasing age, the ability of the body's cells to produce and secrete interleukins decreases, and cellular immunity worsens. In addition, decreased physiological function, increased prevalence of chronic complications, and poor self-care in older patients are associated with longer healing times for foot ulcers [28]. The older the patient, the slower the wound healing time [29]. The Wagner grading method is the most widely used clinical grading method for diabetic foot ulcers, which is based on the depth of the ulcer to classify the diabetic foot into 0–5 levels [30]. Wagner grading directly affects the prognosis of diabetic foot: the higher the grading, the more serious the diabetic foot condition is, the more difficult the treatment is, which increased the wound healing time [31]. C-peptide is a reliable indicator of pancreatic β-cell function [32], a biologically active peptide that activates a variety of cell signaling pathways by binding to cell membrane surface signaling molecules, exerting antioxidant, anti-apoptotic, and regulating inflammatory responses [33, 34], with functions such as protecting blood vessels, preventing endothelial cell death, controlling vascular inflammation, reducing microvascular permeability, and preventing neointima formation [35], which helps promote healing of diabetic foot ulcers. The results of this study showed that patients in the healed group had slightly higher fasting C-peptide levels than those in the non-healing group, which is consistent with the findings of Lin et al. [36]. Fasting C-peptide levels are a protective factor for ulcer healing. HDL-C, an anti-atherosclerotic factor, has long been considered the "good cholesterol" with antioxidant, anti-inflammatory, anti-thrombotic, anti-infective, cytoprotective, and vasodilatory effects that improve tissue blood supply to improve the prognosis of patients with foot ulcer wounds and promote wound healing [37]. However, HDL may lose its original function in many inflammatory and pathological conditions [37–39]. The results of this study showed that patients in the healed group had slightly lower HDL-C results than those in the unhealed group, which is possibly due to the patient's inherent diabetic foot ulcer pathology that affects the function of HDL. It is worth to mention that diabetes personals' wound healing could also be affected by decreased microperfusion, structural protein breakdown, and reduced neutrophil chemotaxis and phagocytosis. For example, it has been shown that VSD therapy could increase would healing through promoting granulation and angiogenesis partly by increasing expression of vascular endothelial growth factor (VEGF) in diabetic foot ulcer [7]. Moreover, it has reported that increased phagocytosis could promote ulcer healing through upregulated cleaning up of the dead or damaged cells and dead bacteria [8]. All these factors should be taken into consideration in the treatment of diabetic foot ulcers.

There are some limitations of this study that should be considered. Firstly, the sample size included in this study was small and the study population comprised only patients with type 2 diabetes-related foot ulcers. Secondly, this study was a retrospective study. Therefore, future multicenter prospective studies with expanded sample sizes and ensuring balanced sample distribution are needed to validate the efficacy and safety of the customized VSD technique and to explore in greater depth the factors that affect the wound healing of foot ulcers in patients with diabetes, in order to guide clinical practice and provide more comprehensive, effective, and cost-effective treatment and care for patients with diabetic foot ulcers.

## Conclusion

In short, this study demonstrates that customized VSD is easy to be accessed at a lower cost and can promote the healing of diabetic foot ulcers compared to VSD. In addition, customized VSD also helps to reduce patients’ financial burden through reducing the cost of the treatment and shortening the hospitalization time.

## Data Availability

The datasets used and/or analyzed during the current study are available from the corresponding author on reasonable request.
